# Hemorrhagic Fever with Renal Syndrome, Zibo City, China, 2006–2014

**DOI:** 10.3201/eid2202.151516

**Published:** 2016-02

**Authors:** Ling Wang, Tao Wang, Feng Cui, Shen-Yong Zhai, Ling Zhang, Shu-Xia Yang, Zhi-Qiang Wang, Xue-Jie Yu

**Affiliations:** Zibo Center for Disease Control and Prevention, Zibo City, China (L. Wang, T. Wang, F. Cui, S.-Y. Zhai, L. Zhang, S.-X. Yang);; Shandong Province Center for Disease Control and Prevention, Jinan City, China (Z.-Q. Wang);; Shandong University, Jinan (X.-J. Yu);; University of Texas Medical Branch, Galveston, Texas, USA (X.-J. Yu)

**Keywords:** hemorrhagic fever with renal syndrome, hantavirus, rodent, zoonoses, China, viruses

## Abstract

Analysis of hemorrhagic fever with renal syndrome cases in Zibo City, China, during 2006–2014 showed that it occurred year-round. Peaks in spring and fall/winter were caused by Hantaan and Seoul viruses, respectively. Rodent hosts were the striped field mouse for Hantaan virus and the brown rat and house mouse for Seoul virus.

Hemorrhagic fever with renal syndrome (HFRS) is caused by hantavirus and transmitted primary by rodents (*1*,*2*). HFRS occurs worldwide, but ≈90% of HFRS cases have been reported in China (*3*). During 2006–2012, a total of 77,558 HFRS cases, including 866 deaths, were reported in China (*4*). Zibo City in Shandong Province, located in eastern China, has a high incidence rate of HFRS (*5*,*6*). We analyzed the clinical data for HFRS cases and surveyed the prevalence of hantaviruses in rodent populations in rural area of Zibo City during 2006–2014.

## The Study

**Figure 1:**
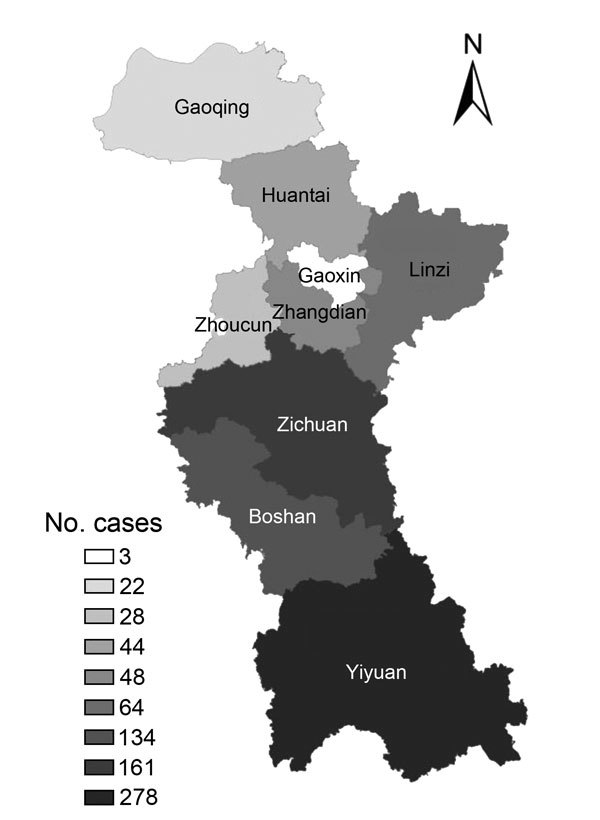
Geographic distribution of cases of hemorrhagic fever with renal syndrome among districts and counties, Zibo City, China, 2006–2014.

Zibo City is a prefecture-level city located at 36°47′N 118°3′E (Figure 1). The city consists of 6 districts and 3 counties distributed over 5,938 km^2^ of land; the total population during the 2010 census was 4.53 million, of whom 900,000 persons were farmers. The city is ≈42% mid-sized mountains in the south, 30% hills in the center, and 28% plains in the north (http://115.238.252.51:9001/html/2007/09/13/20070913141750.html).

We obtained data for HFRS patients from the Zibo Center for Disease Control and Prevention and analyzed these data with Excel 2007 (Microsoft, Redmond, WA, USA) and SPSS 16.0 software (SPSS Inc., Chicago, IL, USA) and χ^2^ test for statistical analysis. The research protocol was approved by the human bioethics committee of the Zibo Center for Disease Control and Prevention, and all participants provided written informed consent. HFRS cases were confirmed by detection of hantavirus IgM in patients’ serum with an ELISA kit (Beijing Wantai, Beijing, China).

During 2006–2014, a total of 782 HFRS cases were reported in Zibo City; 9 (1.2%) persons died. The incidence rate of HFRS varied from 0.74 to 3.65 per 100,000 persons annually (average 1.96 cases/100,000 persons) (Table). HFRS patients were from rural areas in all 9 districts and counties in Zibo City, but most (73.3%) were from hilled areas in the central and southern parts of the city, including Yiyuan County (278 cases), Zichun District (161 cases), and Boshan District (134 cases) (Figure 1). During 2006–2010, HFRS patients were predominantly from Zichuan and Boshan Districts (51.8%) in the central part of the city. During 2011–2014, Yiyuan County became the major source of HFRS cases, accounting for 51.3% of all cases during this period.

**Table:** Characteristics of hemorrhagic fever with renal syndrome, Zibo City, China, 2006–2014

Year	No. cases	Incidence rate, cases/100,000 population	No. deaths (%)	Cases in spring, no. (%)	Cases in fall/winter, no. (%)
2006	156	3.65	0	64 (41.0)	24 (15.4)
2007	90	2.10	0	31 (34.4)	24 (26.7)
2008	69	1.60	1 (1.45)	19 (27.5)	29 (42.0)
2009	35	0.81	1 (2.86)	14 (40)	5 (14.3)
2010	32	0.74	1 (3.13)	6 (18.8)	7 (21.9)
2011	42	0.93	0	5 (11.9)	18 (42.9)
2012	91	2.00	4 (4.40)	12 (13.2)	67 (73.6)
2013	136	2.97	2 (1.53)	35 (25.7)	45 (33.1)
2014	131	2.85	0	44 (33.6)	30 (22.9)
Total	782	1.96	9 (1.15)	230 (29.4)	249 (31.8)

**Figure 2:**
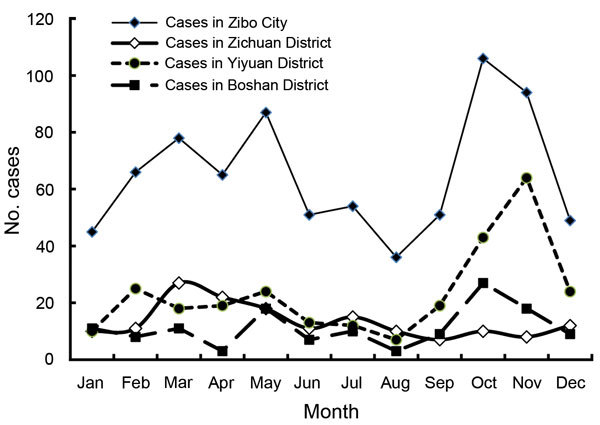
Monthly distribution of cases of hemorrhagic fever with renal syndrome, Zibo City, China, 2006–2014.

HFRS cases peaked twice each year. The spring peak occurred during March–May, and the fall/winter peak occurred during October–December (Figure 2). Serum samples from 36 patients from 2006 through 2008 were typed by focus reduction neutralization test with Hantaan virus (HTNV) strain 76-128 and Seoul virus (SEOV) strain UR in Vero cells. Reducing 80% of plaques was considered a positive result (*7*). Focus reduction neutralization test showed that 93.4% (15/16) patients from the spring peak were infected with SEOV, and 85% (17/20) from the fall/winter peak were infected with HTNV.

The incidence for the fall/winter peak was higher than that for the spring peak for the entire city (Figures 1, 2). However, the seasonal distribution of HFRS cases varied from place to place. For example, Yiyuan County and Boshan District had a substantially higher fall/winter peak of HFRS but did not have an obvious spring peak, whereas in Zichuan District, the spring peak was higher than the fall/winter peak. The number of HFRS cases in the spring peak and fall/winter peak in the 3 high-incidence areas differed significantly (χ^2^ = 74.40, p<0.0001).

The seasonal distribution of HFRS also varied in different years (Table; Figure 1, 2). In 2006, the spring peak was higher than the fall/winter peak, and 41.0% (64/156) of cases reported during that year occurred during March–May. In 2012, one primary peak occurred in which 73.6% (67/91) of cases occurred during the winter. In 2007 and 2010, the peak was not obvious in the spring or the fall. In 2009 and 2014, the spring peak was predominant, and the fall/winter peak was not obvious. In 2008, 2011, and 2013, the epidemic mainly occurred in the fall/winter, when incidence was much higher than for the spring peak. The number of HFRS cases differed significantly between the spring season and fall season each year (χ^2^ = 38.01, p<0.0001).

Ages of the 782 HFRS patients ranged from 7 to 85 years (median 46 years). Most (73.8%) patients were 30–65 years of age, and more than half (51.4%) were 40–50 years of age. Most (68.4%) patients were farmers, and most (87.6%, 685/782) lived in the countryside. The male:female ratio was 2.2:1.

Of the 9 persons who died, 4 died in October, 3 died in November, and 1 each died in August and September. Five decedents were male. Decedent ages ranged from 33 to 60 years; 3 were 30–49 years of age, and 6 were 50–59 years of age. HFRS was diagnosed 3–8 days (average 5.3 days) after illness onset. The patients died 4–8 days (average 5.9 days) after illness onset.

During 2006–2014, we trapped 559 rodents with 21,384 snap-traps in residential area (inside and outside farmhouse) and in fields. The rodents’ lungs were tested for hantavirus antigen with monoclonal antibody (Shanghai Institute of Biologic Products, Shanghai, China) by direct immunofluorescence assay (*8*,*9*). We detected hantavirus antigens in 29 (5.2%) rodents. In residential areas, the brown rat (*Rattus norvegicus*) was the predominant rodent species and had the highest infection rate (5.3%, 21/393). The house mouse (*Mus musculus*) was next in both population and infection rate (2.4%, 1/42). Other residential rodents were all negative for hantavirus, including 16 Chinese hamsters (*Cricetulus griseus*) and 1 striped field mouse (*Apodemus agrarius*). Among field rodents, the striped field mouse was the most common species and had the highest hantavirus infection rate (11.9%, 7/59). Other field rodent species were all negative to hantavirus, including 27 brown rats, 18 buff-breasted rats (*R. flavipectus*), and 3 house mice. Infection rates for the striped field mouse, brown rat, and house mouse did not differ significantly (χ^2^ = 5.50, p = 0.06). The hantavirus antigen–positive rodents were further tested by reverse transcription PCR amplification of hantavirus M segment (*10*) from lung tissue, and 19 rodents were PCR positive. BLAST analysis (http://blast.ncbi.nlm.nih.gov/Blast.cgi) indicated that 13 sequences belonged to SEOV and 6 belonged to HTNV. SEOV-positive rodents were all brown rats from residential areas, and HTNV-positive rodents were all striped field mice from field areas. The reverse transcription PCR–positive rate was 3.3% (13/393) for SEOV in brown rats from residential areas and 10.2% (6/59) for HTNV from field areas.

## Conclusions

HFRS consistently occurred in Zibo City and peaked twice: in the spring and fall/winter. The spring peak was caused mainly by SEOV and the fall/winter peak mainly by HTNV. The major animal host of HTNV was the striped field mouse, and the major animal hosts of SEOV were the residential brown rat and house mouse. The house mouse is not considered the animal host of HTNV or SEOV, but this study and recent studies (*11*–*13*) have detected both HTNV and SEOV in this rodent. The role of the house mouse as the animal host of HTNV and SEOV needs further investigation.
